# Identifying the thresholds of site condition and stand structure for optimizing multiple ecosystem services in the Liupan Mountains, Northwest China

**DOI:** 10.3389/fpls.2026.1970073

**Published:** 2026-10-28

**Authors:** Xiaocha Wei, Yipeng Yu, Pengtao Yu, Yanhui Wang, Xiao Wang, Yanfang Wan

**Affiliations:** Key Laboratory of National Forestry and Grassland Administration on Forest Ecosystem Projection and Restoration, Ecology and Nature Conservation Institute, Chinese Academy of Forestry, Beijing, China

**Keywords:** comprehensive benefit, multiple ecosystem services, site condition, stand structure, thresholds, trade-off

## Abstract

Sustainable forest management is critical for promoting multiple ecosystem services (ESs), yet the thresholds of site condition and stand structure on the comprehensive benefit of multiple ESs remain unclear across forest types at regional scales. We took the Liupan Mountains as a case study and evaluated four key ESs (carbon storage, timber production, water yield, and soil conservation) using biomass models, timber volume equations, and the InVEST model at pixel scale. We then developed a comprehensive index (CI) integrating supply and trade-off indices to assess multiple ES benefits, and quantified the constraining effects and thresholds of site condition and stand structure on CI. The results showed that natural forests provided higher carbon storage, water yield, and soil conservation than planted forests, except for timber production. The supply index (SI), trade-off index (TI), and CI exhibited clear spatial patterns, with higher values in the south and lower values in the north. The SI (0.46 vs. 0.28), TI (0.24 vs. 0.23), and CI (0.89 vs. 0.83) showed differences between natural and planted forests. Mean annual precipitation, stand age, tree height, and DBH showed significant positive correlations with CI, while stand density had a strong negative effect—all with nonlinear relationships and critical thresholds. Thresholds for planted forests (mean annual precipitation = 590 mm, stand age = 28 years, stand density = 1,524 trees/ha, tree height = 12 m, DBH = 14 cm) differed from those of natural forests (651 mm, 54 years, 405 trees/ha, 21 m, 36 cm). Under similar precipitation and stand age, planted forests could be thinned toward natural forest density benchmarks to enhance their comprehensive benefits. Our findings provide regional-scale evidence-based forest management practices for achieving high comprehensive benefits of multiple ESs.

## 1 Introduction

Forests provide multiple ecosystem services (ESs), including timber production, water yield, carbon storage, and soil conservation, all of which are critical for ecological security, human survival, and regional sustainable development (Millennium Ecosystem Assessment (Program), 2005; Costanza et al., 1997; Pukkala, 2016; Brockerhoff et al., 2017). However, the relationships among different ESs are complex, and growing evidence indicates that enhancing one service may lead to the decline of others (Bradford and D'Amato, 2012; Xu et al., 2024). For instance, the Loess Plateau located in the arid and semi-arid regions of northwest China, has historically suffered from severe soil erosion driven by intensive human activities. Following a series of ecological restoration projects implemented between 2001 and 2020, the restored forestland in this region accounted for 7.4% of its total land area (Xue and Lu, 2022). However, limited precipitation has meant that extensive vegetation restoration has adversely affected water yield service (Feng et al., 2016; Pan et al., 2013; Yu et al., 2023). Moreover, the past strict logging ban policy has increased the forest coverage and timber stock, but negatively impacted timber quality, restricted natural regeneration, soil desiccation, and increased stand instability against wind or snow break (Wang and Shao, 2004; Hao et al., 2012; Wang et al., 2015; Chaudhary et al., 2016). These problems indicate that the previous single-objective management model, which focused exclusively on timber production, is no longer appropriate. Therefore, it is both important and urgent to transition toward a multi-objective management systemfor more balanced provision of multiple ESs, while also ensuring forest health and stability and meeting increasing diverse social demands (Wang et al., 2017; Yang et al., 2018; Nelson et al., 2009).

Comprehensive assessment of the benefit of multiple ESs can provide critical information for ecosystem management and policy-making. Several studies have proposed comprehensive index that integrate multiple ESs into a single metric to represent their overall value. For instance, Pan et al. (2013) developed the Total Ecosystem Services (TES) index to quantify the spatial dynamics of multiple ESs supply, while Lang and Song (2019) proposed the Ecosystem Services index (ESI) to evaluate the degree of variation in multiple ESs. However, these approaches, like many others, simply calculate the total or average value of multiple ESs with equal or differential weighting (Shen et al., 2021; Wu and Fan, 2022). Although these methods are straightforward and effective for measuring overall ES performance, they generally fail to account for trade-offs among multiple ESs (Ouyang et al., 2016). For those existing studies that have accounted for ES trade-offs, they have typically evaluated them by comparing the magnitude of the temporal variation among services using correlation coefficients, but they have failed to quantify the intensity of individual trade-offs (Yang et al., 2018). Bradford and D'Amato (2012) proposed a simple approach to quantify the outcomes of alternative management options in terms of both benefits and trade-offs across multiple objectives, using the mean value and root mean square error of multiple ESs. Nevertheless, few studies have comprehensively evaluated ES performance by simultaneously considering both the supply capacity and trade-offs among different ESs. Furthermore, Wang et al. (2022) proposed an ES index that incorporates both supply capacity and trade-off effects to reflect the complex interactions among multiple ESs, and identified a threshold of forest proportion based on this index, providing a useful reference for determining appropriate forest coverage. However, forest management practice, knowing the optimal forest proportion alone is insufficient. While these studies have identified stand density, stand age, elevation, and slope aspect as key drivers of individual ESs, most were conducted at the site or slope scale and focused on one or a few services. It therefore remains to be determined whether the structural thresholds identified at these scales hold at regional scale, and how they vary across forest types and site conditions when multiple ESs and their trade-offs are considered simultaneously.

Due to spatial heterogeneity in driving factors (e.g., vegetation, climate, topography, and soil), the supply of multiple ESs and their complex interactions vary across forest types (e.g., coniferous, broad-leaved, mixed, natural and planted forests) (Dai et al., 2022). A previous study found that planted forests exhibit higher hydraulic safety but lower hydraulic efficiency than natural forests at the species level, and natural forests show a trade-off between hydraulic efficiency and safety, whereas planted forests display a contrasting positive correlation between these traits (Bai et al., 2026). Even within the same forest type, ES dynamics differ under varying stand structure and site condition. Recent studies have identified stand density, stand age, and elevation as primary factors influencing leaf area index, evapotranspiration, and water yield (Tian et al., 2021), and have shown that stand transpiration is higher in shady slopes than on sunny slopes (Liu et al., 2024). According to the review of Forrester (2019) stand structure (stand density, tree size and size distributions) can strongly influence forest growth and related processes, including water balance, carbon allocation, nutrient cycling, and light dynamics. Felipe-Lucia et al. (2018) found that managing forests to increase structural heterogeneity, maintain large trees, and create canopy gaps would promote the supply of multiple ESs simultaneously. However, most of these studies have been conducted at the site or slope scale, which substantially limits their applicability for guiding regional or basin scale forest management decisions on how to enhance the comprehensive benefits of multiple ESs under varying site condition and stand structure. Although global forest area and timber volume have increased considerably following a series of reforestation and afforestation efforts, there is now an urgent need to optimize the stand structure to improve the forest quality and strengthen the overall benefits of multiple ESs. Therefore, analyzing the effect of site condition and stand structure on the comprehensive benefits of multiple ESs at regional scale is critical for informing forest management practices.

In addition, the effects of driving factors on ESs are often characterized by complex nonlinear relationships, which remain relatively stable within a given range but may trigger abrupt changes in ecosystem functions once thresholds are exceeded, potentially leading to irreversible and undesirable consequences (Asbjornsen et al., 2022). Threshold responses of forest cover to hydrological regulation are particularly critical for ecosystem management. For example, restoring at least 20% of the watershed to tropical montane cloud forest is required to achieve a significant increase in streamflow (Teixeira et al., 2021). Similarly, Wang et al. (2022) found that increasing forest cover does not consistently improve the comprehensive index, as it reaches its maximum only when the forest proportion attains a critical threshold. Threshold behavior can also manifest temporal scales. For instance, Liu et al. (2024) reported that to maintain high biodiversity and stand volume, the optimum stand density for stand age of 30, 40, 50, and 60 years are 1,390, 1,153, 1,042, and 871 trees/ha, respectively. Ahmad et al. (2019) found that an optimal stand density of approximately 0.7 should be maintained to sustain both high species diversity and adequate timber production. Similarly, Hao et al. (2012) suggested that a stand density range of 1,000-1,200 tree/ha could balance multiple ESs. These studies have provided valuable evidence of threshold behavior in forest-hydrology and structure-diversity relationships. However, they are largely based on plot-level field observations, and the thresholds reported are specific to particular stand ages, species, or sites. Consequently, it is still unclear (i) whether comparable thresholds exist for the comprehensive benefits of multiple ESs, (ii) how such thresholds shift across different forest types and site conditions, and (iii) whether they can be upscaled to guide regional forest management. Therefore, to better promote the provision of multiple ESs while minimize trade-offs among different management objectives, it’s essential to investigate the constraining effects and critical thresholds of site conditions and stand structure on the supply capacity of multiple ESs, and their complex interactions across different forest types at a regional scale. Such knowledge can directly support forest managers in designing regional-specific management strategies.

Therefore, taking the Liupan Mountains (LPM) as a case study, we evaluated four key ESs (carbon storage, timber production, water yield, soil conservation) using the biomass model, timber volume model, InVEST model, and USLE model. We then assessed the comprehensive benefits of multiple ESs using a comprehensive index that integrates a supply index and a trade-off index, and ultimately quantified the constraining relationships and identified the thresholds of key drivers affecting on the comprehensive index. The specific objectives of this study were: (1) to evaluate the spatial heterogeneity of four individual ESs and their variation across different forest types; (2) to quantify the comprehensive benefits of multiple ESs using a comprehensive index; and (3) to identify the thresholds of site condition and stand structure on the comprehensive index for both natural and planted forests. We anticipate that our findings will provide researchers, stakeholders and forest managers with a reference for developing evidence-based strategies to optimize stand structure to achieve high-level comprehensive benefits of multiple ESs.

## 2 Materials and methods

### 2.1 Study area

The Liupan Mountains (LPM), located in the Northwest of China (106.18-106.50^°^E, 35.23-35.94^°^N), cover an area of 898 km^2^ (**Figure 1a**) with the elevations ranging from 1,612 to 2,942 m (**Figure 1b**). The soil types are mainly Haplic Greyzems at the elevations between 1,700 m and 2,700 m, and Gelic Cambisols above 2,600 m, with soil thickness varying from 30 cm in south to 150 cm in north (FAO/IIASA/ISRIC/ISSCAS/JRC, 2012). This region has a semi-arid to semi-humid temperate climate, with a mean annual precipitation ranging from 478 mm in the north to 684 mm in the south. The mean annual temperature is 5.8 °C, with a range of 2.4 - 8.3 °C and decrease from the north to the south of LPM due to rising elevation.

**Figure 1:**
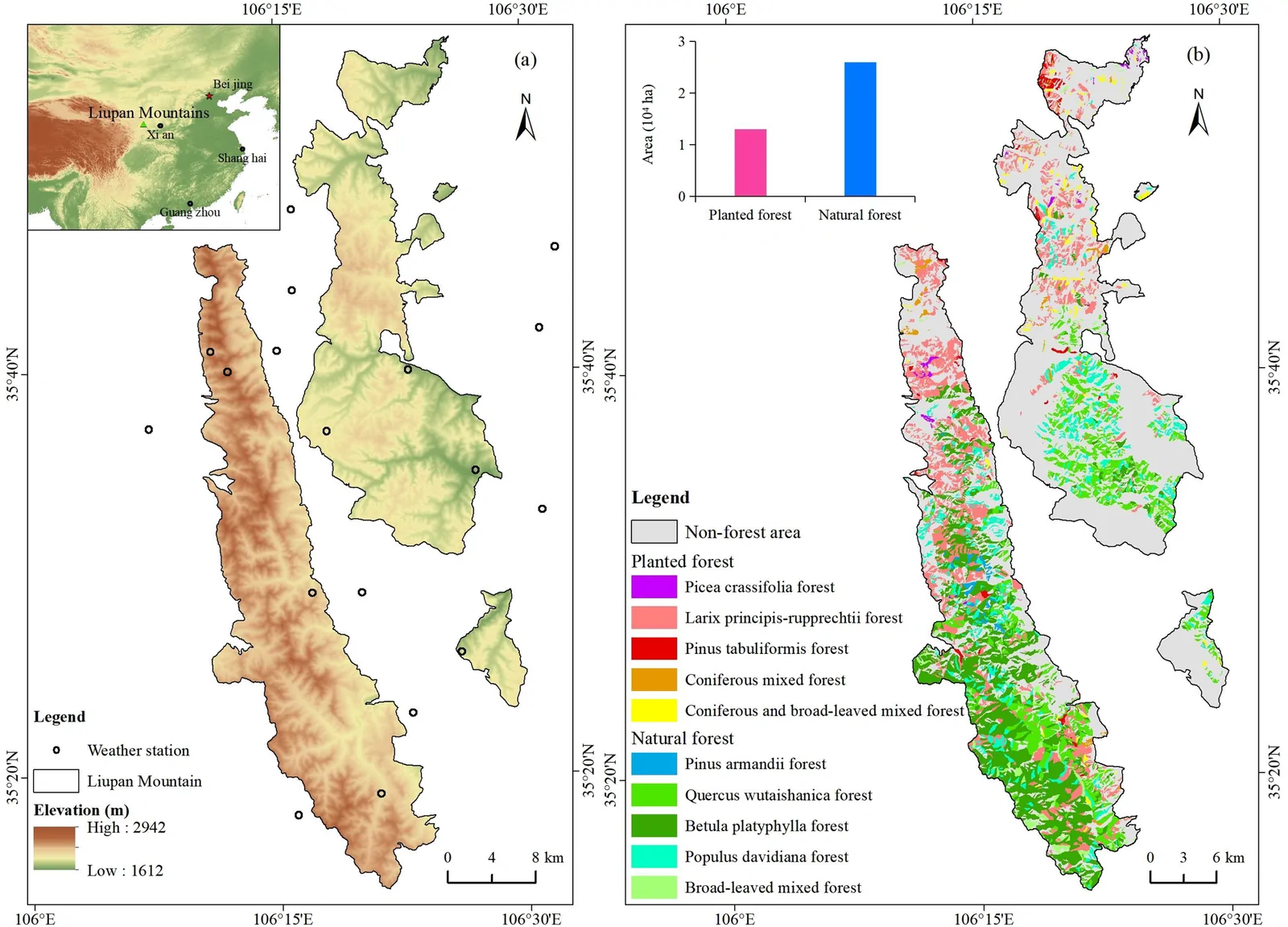
The location **(a)** and forest types **(b)** of the Liupan Mountains.

The vegetation is mainly composed of temperate deciduous broad-leaved forest, coniferous-broad-leaved mixed forest, mountain shrub grassland, mountain grassland, sub-alpine meadow. Forest resources are abundant, with a total forest area of 55,921 ha, accounting for 62% of LPM’s total area. Natural forests, which are predominantly *Betula platyphylla* forest, *Quercus wutaishanica* forest, and *Populus davidiana* forest, occupy 67% of the total arboreal forest area (39,024 ha) and are mainly distributed in the southern part of LPM. These natural forests are characterized by uneven-aged, multilayered, moderate density, rich species composition, high biodiversity, complex structure, stable ecosystems, and strong resistance to disturbances. Their average stand age, stand density, canopy density, tree height, diameter at breast height (DBH), and leaf area index (LAI) are 40 years, 1,173 trees/ha, 0.45, 9.2 m, 15.1 cm, 3.0.

Over past several decades, large-scale afforestation and reforestation have been carried out in the LPM to reduce soil erosion and increase timber production (Wang et al., 2008; Yu et al., 2009). As a result, extensive planted forests, mainly plantations of *Larix principis-rupprechtii*, *Pinus tabuliformis* and *Picea crassifolia*, now occupy the arid northern part of the LPM. In contrast to natural forests, these planted forests are largely even-aged and pure stands, with simple vertical structure, sparse understory vegetation, high density, low species diversity, fragile ecosystems, and poor resilience to disturbances. Their average characteristics are as follows: stand age of 26 years, stand density of 1,512 trees/ha, canopy density of 0.43, tree height of 10.8 m, DBH of 12.0 cm, and LAI of 3.2.

### 2.2 Methods

#### 2.2.1 The ecosystem service evaluation

Total carbon density was used to evaluate the carbon storage service (CS, t/ha, Equation 1) of different forest types at pixel scale. It is the sum of carbon density from five components: the arboreal layer (CD_arbor_, Equations 2, 3), shrub layer (CD_shrub_, Equation 4), herbal layer (CD_herb_, Equation 5), litter layer (CD_litter_, Equation 6), and the soil layer (0–100 cm) (CD_soil_, Equations 7–9).

(1) $$\text{CS}={\text{CD}}_{\text{arbor}}+{\text{CD}}_{\text{shrub}}+{\text{CD}}_{\text{herb}}+{\text{CD}}_{\text{litter}}+{\text{CD}}_{\text{soil}}$$

(2) $${\text{CD}}_{\text{arbor}}=\left({\text{B}}_{\text{arbor}}\times \text{STD}÷1000\right)\times {\text{R}}_{\text{arbor}}$$

(3) $${\text{B}}_{\text{arbor}}=\exp\left(\text{a}\times \ln\left({\text{DBH}}^{\text{b}}\times {\text{H}}^{\text{c}}\right)-\text{d}\right)$$

(4) $${\text{CD}}_{\text{shrub}}=0.177\times \exp\left(-9.08\times 10^{-5}\times {\text{LAI}}^{6.41}+4.65\right)\times {\text{R}}_{\text{shrub}}$$

(5) $${\text{CD}}_{\text{herb}}=0.28\times \exp\left(-0.12278\times {\text{LAI}}^{0.2}+1.37\right)\times {\text{R}}_{\text{herb}}$$

(6) $${\text{CD}}_{\text{litter}}=\left(0.1038\times {\text{B}}_{\text{arbor}}+10.901\right)\times {\text{R}}_{\text{little}}$$

(7) $${\text{CD}}_{\text{soil}}=\left(1.11-0.010\times \text{AMT}-0.024\times \text{DEM}\right)\times {\text{SOCD}}_{0}\times \exp\left(-0.011\times \text{AGE}\right)+0.079\times {\text{CD}}_{\text{arbor}}\times \exp\left(0.035\times \text{AGE}\right)$$

(8) $${\text{SOCD}}_{0_{\text{P}}}=\left(-0.002\times {\text{DEM}}^{2}+8.241\times \text{DEM}-6191.891\right)\times \left(-0.00000187\times {\text{MAP}}^{2}+0.00161\times \text{MAP}-0.214\right)$$

(9) $${\text{SOCD}}_{0_{\text{N}}}=\left(-2.58\times 10^{-11}\times {\text{DEM}}^{2}+1.22\times 10^{-7}\times \text{DEM}-0.00012\right)\times \left(0.0002\times {\text{ASP}}^{2}-0.025\times \text{ASP}-7.206\right)\times \left(-4.161\times {\text{MAT}}^{2}+40.387\times \text{MAT}-399.429\right)\times \left(-5.25\times {\text{AGE}}^{2}+449.676\times \text{AGE}-1977.387\right)$$

where B_arbor_ is the arboreal layer biomass of single tree (kg/tree), with the biomass model parameters (a, b, c, d) and carbon ratios of arboreal layer (B_arbor_) provided in **Table 1** (Pan et al., 2014); STD is stand density (trees/ha); DBH is average tree diameter at breast height (cm); H is average tree height (m); LAI is leaf area index; R_shrub_ = 0.4781 is the carbon ratio of shrub layer; R_herb_ = 0.3692 is the carbon ratio of herbaceous layer; R_little_ = 0.3815 is the carbon ratio of little layer; MAT is mean annual temperature (°C); DEM is elevation (m); AGE is stand age (year); MAP is mean annual precipitation (mm); ${\text{SOCD}}_{0_{\text{P}}}$ and ${\text{SOCD}}_{0_{\text{N}}}$ are the initial soil carbon density of planted and natural forests; ASP is slope aspect (°). The carbon density equation of shrub layer, herbal layer and litter layer were adopted from Tian (2019), and the carbon density equation of soil layer from Zhang et al. (2021).

**Table 1:** The parameter of arboreal layer biomass model and carbon ratio in different forest types used in Equations 2 and 3.

Forest types	a	b	c	d	R_arbor_	Forest types	a	b	c	d	R_arbor_
*Picea crassifolia* forest	2.3307	1	0	2.0770	0.522	*Pinus armandii* forest	2.4119	1	0	2.2962	0.4926
*Larix principis-rupprechtii* forest	2.4419	1	0	2.3442	0.4903	*Quercus wutaishanica* forest	0.9979	2	1	3.5426	0.522
*Pinus tabuliformis* forest	2.3803	1	0	2.3503	0.522	*Betula platyphylla* forest	0.8114	2	1	1.8989	0.4837
Coniferous mixed forest	0.9037	2	0	2.5700	0.522	*Populus davidiana* forest	0.9222	2	1	2.8360	0.503
Coniferous and broad-leaved mixed forest	0.9037	2	0	2.5700	0.522	Broad-leaved mixed forest	0.9037	2	1	2.5700	0.522

Timber volume was used to evaluate the timber production service (TP, m^3^/ha) of different forest types at the pixel scale. The parameters of timber volume model were adopted from previous studies (Liupanshan Forestry Bureau, 1988; Li et al., 2013; Zeng, 2018; Tian, 2019), as shown in **Table 2**.

**Table 2:** The parameter of timber volume model of different forest types used in Equation 10.

Forest types	e	f	g	Forest types	e	f	g
*Picea crassifolia* forest	0.000053108582	1.778667	1.1280516	*Pinus armandii* forest	0.000059973839	1.8334312	1.0295315
*Larix principis-rupprechtii* forest	0.00003534	2.357	0.7	*Quercus wutaishanica* forest	0.000060970532	1.8735078	0.94157465
*Pinus tabuliformis* forest	0.000066492455	1.8655617	0.93768879	*Betula platyphylla* forest	0.000052286055	1.8598021	1.01401715
Coniferous mixed forest	0.000057887451	1.8445849	0.98088457	*Populus davidiana* forest	0.000054031091	1.9440215	0.93067368
Coniferous and broad-leaved mixed forest	0.000057887451	1.8445849	0.98088457	Broad-leaved mixed forest	0.000057887451	1.8445849	0.98088457

(10) $$\text{TP}=\text{e}\times {\text{DBH}}^{\text{f}}\times {\text{H}}^{\text{g}}\times \text{STD}$$

where e, f, and g are dimensionless constants and shown in **Table 2**.

Water yield depth was used to evaluate the water yield service (WY, mm/yr) of different forest types at the pixel scale (Yang et al., 2020). Based on the water balance equation, the InVEST 3.10.2 Anual Water Yield module was used to quantify water yield, following the methodology described in the InVEST User’s Guide (Natural Capital Project, 2022). The calculation formulas are as follows:

(11) $$\text{WY}=\left(1-\frac{\text{AET}}{\text{AMP}}\right)\times \text{AMP}$$

(12) $$\frac{\text{AET}}{\text{AMP}}=\frac{1+\omega \times \text{R}}{1+\omega \times \text{R}+\frac{1}{\text{R}}}$$

(13) $$\text{R}=\frac{{\text{K}}_{\text{c}}\times {\text{ET}}_{0}}{\text{P}}$$

(14) $$\text{Kc}=\{\begin{array}{cc} \frac{\text{LAI}}{3}\; & \text{LAI}\leq 3\\ 1 & \text{LAI}>3 \end{array}$$

(15) $$\omega =\text{Z}\times \frac{\text{AWC}}{\text{MAP}}$$

(16) Z=0.2×N

(17) $$\text{AWC}=\text{Min}\left({\text{Depth}}_{\text{soil}}\;,{\text{Depth}}_{\text{root}}\right)\times \text{PAWC}$$

where AET is the actual evapotranspiration (mm); AMP is the annual mean precipitation (mm); R is the aridity coefficient; *ω* is a non-physical parameter characterizing the natural climatic-soil properties; K_c_ is the evapotranspiration coefficient; ET_0_ is potential evapotranspiration (mm); Z is the seasonality factor; N is the number of rainfall events with duration exceeding 6 hours, which was recorded as 37 events at the Jingyuan meteorological station; AWC is the volumetric plant-available water content; PAWC is the plant-available water capacity, set to 80% of filed capacity; Depth_soil_ is the soil layer depth (cm); and Depth_root_ is vegetation root depth (cm).

To validate the accuracy of the annual water yield service estimated by InVEST model, we compared our simulated runoff coefficients with values reported in published studies from comparable forest ecosystems in nearby regions. Specifically, the simulated runoff coefficient was 0.17 for *Picea crassifolia* forest (while the observed was 0.20, meaning a relative error of -12.71%), 0.16 for *Larix principis-rupprechtii* forest (observed of 0.16, relative error of 1.85%), 0.16 for *Pinus tabuliformis* forest (observed of 0.20, relative error of -18.29%), 0.21 for coniferous mixed forest (observed of 0.19, relative error of 11.87%), 0.18 for mixed forest (observed of 0.19, relative error of -4.78%), 0.19 for *Pinus armandii* forest (observed of 0.23, relative error of -15.80%), 0.14 for *Quercus wutaishanica* forest (observed of 0.17, relative error of -14.99%), 0.21 for *Betula platyphylla* forest (observed of 0.20, relative error of 5.15%), 0.26 for *Populus davidiana* forest (observed of 0.29, relative error of -10.84%), and 0.19 for broad-leaved mixed forest (observed of 0.22, relative error of -13.12%). The simulated runoff coefficients were generally close to the reported values, with relative errors ranging from 1.85% to 18.29%, indicating that the magnitude of our water yield estimates is consistent with independent regional observations and adequate for the purposes of this study.

The soil conservation service is evaluated as the difference between the potential soil erosion and the actual soil erosion (Zhou et al., 2023). Soil erosion was estimated using the Universal Soil Loss Equation (USLE) framework (Wischmeier and Smith, 1978) as implemented in the Sediment Delivery Ratio module of InVEST 3.10.2 (Natural Capital Project, 2022). The soil conservation service (SC, t/ha/yr) is calculated as follows:

(18) SC=R×K×LS×(1−C×P)

(19) $$\text{R}=24\times {\text{R}}_{\text{i}}$$

(20) $${\text{R}}_{\text{i}}=\alpha \times \sum_{\text{j}=1}^{\text{k}}{\left({\text{D}}_{\text{j}}\right)}^{\beta }$$

(21) $$\beta =0.8386+\frac{18.144}{{\text{P}}_{\text{d}12}}+\frac{24.455}{{\text{P}}_{\text{y}12}}$$

(22) $$\alpha =21.586\times \beta^{-7.1891}$$

(23) $$\text{K}=\left\{0.2+0.3\times \exp\left[-0.0256\times {\text{S}}_{\text{d}}\left(1-\frac{{\text{S}}_{\text{i}}}{100}\right)\right]\right\}\times {\left(\frac{{\text{S}}_{\text{i}}}{{\text{C}}_{\text{i}}+{\text{S}}_{\text{i}}}\right)}^{0.3}\times \left[1-\frac{0.25\times {\text{C}}_{\text{or}}}{{\text{C}}_{\text{or}}+\exp\left(3.72-2.95\times {\text{C}}_{\text{or}}\right)}\right]\times \left\{1-\frac{0.7\times \left(1-\frac{{\text{S}}_{\text{d}}}{100}\right)}{\left(1-\frac{{\text{S}}_{\text{d}}}{100}\right)+\exp\left[-5.51+22.9\times \left(1-\frac{{\text{S}}_{\text{d}}}{100}\right)\right]}\right\}$$

(24) $$\text{L}={\left(\lambda /22.13\right)}^{\alpha }$$

(25) $$\alpha =\frac{\beta }{1+\beta }$$

(26) $$\beta =\left(\sin\theta /0.0896\right)/\left[\left(3*\sin\theta^{0.8}\right)+0.56\right]$$

(27) $$\text{S}=\{\begin{array}{cc} 10.8\times \sin\theta +0.03, & \theta <5{}^{\circ}\\ 16.8\times \sin\theta -0.50, & 5\leq \theta <10{}^{\circ}\\ 21.91\times \sin\theta -0.96 & \theta \geq 10{}^{\circ} \end{array}$$

(28) $$\sin\theta =\sin\left(\frac{\theta \times 3.1415926}{180}\right)$$

(29) $$\{\begin{array}{cc} C=1 & \text{VC}=0\\ C=0.6508-0.3436\times \mathrm{lg}\text{VC} & 0<\text{VC}\leq 78.3\%\\ C=0\; & \text{VC}>78.3\% \end{array}$$

(30) $$\text{VC}=\frac{\text{NDVI}-{\text{NDVI}}_{\min}}{{\text{NDVI}}_{\max}-{\text{NDVI}}_{\min}}$$

(31) $$\text{LAI}=0.01065\times {\text{e}}^{9.388\times \text{NDVI}}$$

(32) $$\text{LAI}=5.5\times \left(1-\text{EXP}\left(-0.0767\times {\text{STD}}^{0.0948}\right)\right)\times \text{EXP}\left(-9.937\times 10^{-7}\times {\text{DEM}}^{2}+0.00494\times \text{DEM}-4.548\right)\times \left(1.1\times {\left(1-\text{EXP}\left(-0.154\times \text{AGE}\right)\right)}^{3.2}-0.55\times {\left(1-\text{EXP}\left(-0.1123\times \text{AGE}\right)\right)}^{95.49}\right)$$

Where R is the annual rainfall erosivity parameter (MJ·mm/(ha·h·yr)), K is the soil erodibility parameter (t·ha·h/(MJ·ha·mm)); LS is the slope length-gradient factor; C and P are the vegetation cover factor and soil and water conservation measure factor of different forest types; Since no soil and water conservation measures are implemented in the study area, P is set to 1; k is the number of days in half-month period; D_j_ is the daily rainfall amount (≥12 mm) on the j-th day within that period; P_dl2_ is the average daily rainfall amount (≥12 mm); P_yl2_ is the average annual rainfall amount (≥12 mm); S_d_, S_i_, C_i_, and C_or_ are the contents of sand, silt, clay, and soil organic carbon (%); λ is horizontal projection slope length (m), calculaed as the product of flow accumulation and cell size in ArcGIS; α is variable slope length index; β is the ratio of rill erosion volume to inter-rill erosion volume; θ is slope (°); VC is vegetation coverage (%); NDVI is normalized difference vegetation index; NVDI_min_ and NDVI_max_ are the minimum and maximum NDVI value. Equation 31 is build by Wang (2009) and Equation 32 is build by Tian et al. (2019).

We compared our simulated soil erosion modulus with previous studies conducted in nearby regions. Specifically, the average simulated soil erosion modulus of this study region was 2.46 t/ha/yr, which was similar to the reported value of 2.25 t/ha/yr in the Qinling Mountains (Feng and Han, 1985). And the simulated soil erosion modulus was 3.07 t/ha/yr for *Pinus tabuliformis* forest, very close to the reported value of 3.15 t/ha/yr in the Funiu Mountain Region (Zhang et al., 2020). That means the simulated erosion modulus values were close to the reported values, with small relative differences ranging from approximately -2.5% to 9.3%, indicating that the magnitude of simulated soil-conservation service is consistent with the independent observations and adequate for the purposes of this study.

#### 2.2.2 The comprehensive index of multiple ESs

For a sustainable ecosystem, it is not only important to have a large supply capacity of multiple ESs, but the relationships among multiple ESs also should be synergistic. Therefore, a comprehensive index (CI) was used to evaluate the synergistic supply of multiple ESs by integrating the supply index (SI) and trade-off index (TI) according to previous study (Wang et al., 2022). The calculation equations are as followings:

(33) $$\text{CI}=\sqrt{\left({\text{W}}_{\text{s}}\times {\text{SI}}^{2}+{\text{W}}_{\text{t}}\times {\left(1-\text{TI}\right)}^{2}\right)}$$

(34) $$\text{SI}=\frac{1}{4}\sum_{\text{i}=1}^{4}{\text{ES}}_{{\text{std}}_{\text{i}}}$$

(35) $${\text{ES}}_{{\text{std}}_{\text{i}}}=\frac{{\text{ES}}_{{\text{obs}}_{\text{i}}}-{\text{ES}}_{\min_{\text{i}}}}{{\text{ES}}_{\max_{\text{i}}}-{\text{ES}}_{\min_{\text{i}}}}$$

(36) $$\text{TI}=\text{RMSE}=\sqrt{\frac{1}{3}\sum_{\text{i}=1}^{4}{\left({\text{ES}}_{{\text{std}}_{\text{i}}}-\bar{\text{ES}}\right)}^{2}}$$

(37) $$\text{BI}={\text{ES}}_{{\text{std}}_{\text{i}}}-\bar{\text{ES}}$$

Where W_s_ and W_t_ are weighting coefficients representing the relative importance of SI and TI. We assumed equal important of both, thus setting W_s_ = W_t_ = 1; SI is the supply index representing the supply capacity of four ESs, it can be estimated by taking the mean of the standardized values of the four individual ESs (Bradford and D'Amato, 2012); TI is the trade-off index representing the trade-off magnitude among the four ESs, it was evaluated by calculating the root mean square error (RMSE) of the individual ES, RMSE approximates the average deviation from the mean ES in two dimensions (Bradford and D'Amato, 2012); (1-TI) presents the magnitude of synergistic among the four ESs; Geometrically, SI and (1-TI) can be regarded as the two legs of an isosceles right triangle, representing two vectors that jointly contribute to CI with equal importance. CI increases only when both SI and (1-TI) are high; ${\text{ES}}_{{\text{std}}_{\text{i}}}$ is the standardized value of ES i; ${\text{ES}}_{{\text{obs}}_{\text{i}}}$ is the observed value of ES i; ${\text{ES}}_{\min_{\text{i}}}$ and ${\text{ES}}_{\max_{\text{i}}}$ are the minimum and maximum values of ES i across the entire study area; and $\bar{\text{ES}}$ is the average value of the four ESs, which equals SI; BI is the benefit index.

#### 2.2.3 Identifying the dominant driving factors and analyzing their relationship with CI

Firstly, the correlation analysis was used to identity the dominant drivers of stand structure and site condition influencing the CI. Then, the structural equation modeling (SEM) was employed to analyze their influence pathways. We specified the causal structure among variables directly using the path diagram drawing tool in AMOS 29 software. This structure was established based on the ecological theoretical framework of “site-structure-function,” and the variables were organized into three hierarchical levels: exogenous variables (topographic and climatic factors), endogenous variables (stand structure), and the final response variable (the comprehensive index). Parameters of SEM were estimated using maximum likelihood estimation in AMOS. The df (degrees of freedom) of the model was 48, and the fit indices indicated an acceptable model fit. After that, our objective is to identify the maximum CI under given supply conditions. Therefore, the boundary line method was then employed to construct the constraint relationships between the driving factors and CI. Specifically, the value range of each driver was divided into 100 equal intervals. For each interval, the boundary point was identified as the highest CI value. Those 100 maximum points were then used to fit a upper boundary line, which represents the optimal (unconstrained) state within each CI interval. Finally, the highest value on this fitted line was identified as the critical threshold between drivers and CI, the corresponding CI values indicate the maximum potential performance - rather than a marginal optimum derived from an explicit multi-objective optimization.

### 2.3 Data preparation

The Category II data from the 4th Forest Resource Inventory carried out in 2016 were obtained from the Liupan Mountain Forestry Bureau and classified into 10 forest types following the Classification of Forestry Resources (GB/T 14721-2010). These forest types were further grouped into planted and natural forests. Planted forests included *Picea crassifolia*, *Larix principis-rupprechtii*, *Pinus tabuliformis*, coniferous mixed, coniferous and croad-leaved mixed forests, whereas natural forests comprised *Pinus armandii*, *Quercus wutaishanica*, *Betula platyphylla*, *Populus davidiana* and croad-leaved mixed forests (**Figure 1d**). Stand age, stand density, canopy density, tree height, and DBH (measured at 1.3 m) for each forest type were also extracted from this dataset. Although the inventory represents a single year, forest patches across the study region differ substantially in stand age, stand density, DBH, and forest types. This spatial variation allows us to compare the ES supply and their trade-offs across stands of different ages under a range of site conditions, although it does not capture stand development over time. The original shapefile was converted to raster data at a resolution of 30 m×30 m using ArcGIS software. Elevation, slope gradient, and slope aspect were derived from the ASTER GDEM dataset (ASTGTM), downloaded from the Geospatial Data Cloud (http://www.gscloud.cn/), and extracted by masking with the LPM boundary. Climate data for different forest types were obtained from the China Meteorological Administration (http://data.cma.cn/) and the Guyuan Meteorological Bureau, comprising 23 meteorological stations with records from 1980 to 2018, including daily precipitation, temperature, relative humidity, wind speed, wind direction. Based on these observations and the Penman-Monteith equation (Allen et al., 1998), annual potential evapotranspiration was calculated using the ET_0_ Calculator tool (https://zhuanlan.zhihu.com/p/377392870). These station data were then interpolated to raster format at 30 m×30 m resolution using the Kriging method in ArcGIS, with elevation as a covariate to account for topographic effects on climate. Annual rainfall erosivity and soil erodibility factor datasets were obtained from the National Earth System Science Data Center (http://www.geodata.cn/). To harmonize the temporal differences, we used multi-year averages for each dataset rather than values from a single year, all variables entering the model represent long-term mean conditions. Basic information and application details for all datasets are summarized in **Table 3**.

**Table 3:** The basic information and application of different data set.

Data set	Data source	Index	Resolution	Year	Application
Forest type	The 4^th^ category II inventory of forest resources from the Liupan Mountains Forestry Bureau	Stand density, canopy density, tree height, DBH, soil thickness	30 m×30 m	2016	CS, TP, WY, SC
DEM	Geospatial Data Cloud (http://www.gscloud.cn/)	Elevation, slope, and aspect	30 m×30 m	–	CS WY, SC
Meteorological data	China Meteorological Administration (http://data.cma.cn/) and local Meteorological Bureau of Guyuan	Precipitation, temperature, potential evapotranspiration	30 m×30 m	1980-2018	CS, TP, WY
Other data sets	National Earth System Science Data Center (http://www.geodata.cn/)	Annual rainfall erosivity, soil erodibility	30 m×30 m	1981-2010, 2018	SC

CS, TP, WY, SC are carbon storage, timber production, water yield and soil conservation service.

## 3 Results

### 3.1 Spatial heterogeneity of four individual ESs

Compared with planted forests, natural forests provided higher ES of carbon storage, water yield, and soil conservation but slightly lower ES of timber production (**Figure 2**). On average, natural forests had 344 t/ha of carbon storage, 107 mm/yr of water yield, and 29 t/ha/yr of soil conservation, they were 33%, 33%, and 7% higher than that of planted forests, respectively, whereas timber production (130 m^3^/ha) was 6% lower. Substantial variation in ESs was also observed among natural forest types. *Betula platyphylla* forest, located in the southern LPM at higher elevations with abundant precipitation, delivered the highest carbon storage (385 t/ha), followed by *Quercus wutaishanica* forest, *Pinus armandii* forest, broad-leaved mixed forest and *Populus davidiana* forest, which showed no marked differences and were mainly distributed in the central and northern parts of the study area. For timber production, *Betula platyphylla* forest ranked first (193 m^3^/ha), followed by *Quercus wutaishanica* forest (115 m^3^/ha), whereas *Pinus armandii* forest, *Populus davidiana* forest and broad-leaved mixed forests had lower levels with no significant differences. *Betula platyphylla* forest also produced the highest water yield (123 mm/yr), followed by *Pinus armandii* forest (111 mm/yr), broad-leaved mixed (108 mm/yr), and *Quercus wutaishanica* forest (103 mm/yr), while *Populus davidiana* forest had the lowest (84 mm/yr).

**Figure 2:**
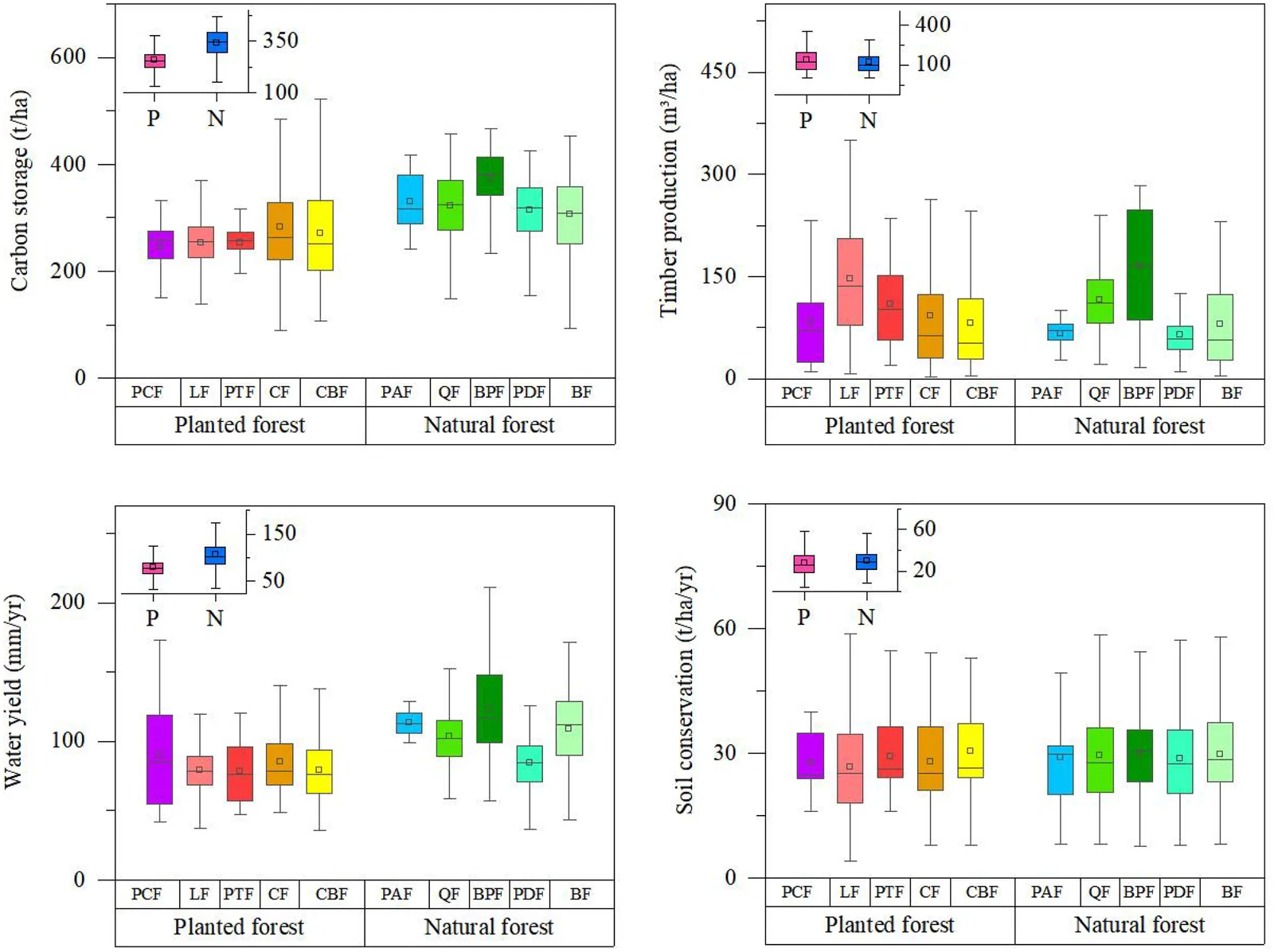
The four individual ESs of different forest types (PCF, *Picea crassifolia* forest; LF, *Larix principis-rupprechtii* forest; *PTF*, *Pinus tabuliformis* forest; CF, Coniferous mixed forest; CBF, Coniferous and broad-leaved mixed forest; PAF, *Pinus armandii* forest; QF, *Quercus wutaishanica* forest; BPF, *Betula platyphylla* forest; PDF, *Populus davidiana* forest; BF, Broad-leaved mixed forest. The same bellow.).

Carbon storage also varied markedly among planted forest types. Coniferous mixed forest, coniferous and broad-leaved mixed forest provide high carbon storage, while *Picea crassifolia* forest, *Larix principis-rupprechtii* forest, and *Pinus tabuliformis* forest exhibited low carbon storage service, all below the levels of natural forests. Owing to its large area, *Larix principis-rupprechtii* forest contributed substantially to timber production, averaging 152 m^3^/ha, a same level comparable to that of *Betula platyphylla* forest. Although *Picea crassifolia* forest, *Pinus tabuliformis* forest, coniferous mixed forest, coniferous and broad-leaved mixed forest provide lower timber production than *Larix principis-rupprechtii* forest, they still outperformed most natural forest types. *Larix principis-rupprechtii* forest provide the lowest water yield, and all other planted forests provide lower water yield than natural forests. Soil conservation showed no marked difference between natural and planted forests, nor among forest types within each category. All four individual ESs exhibited high spatial heterogeneity across the study region, with higher values in the south and lower values in the north; timber production additionally showed a peak in the central-western LPM (**Figure 3**).

**Figure 3:**
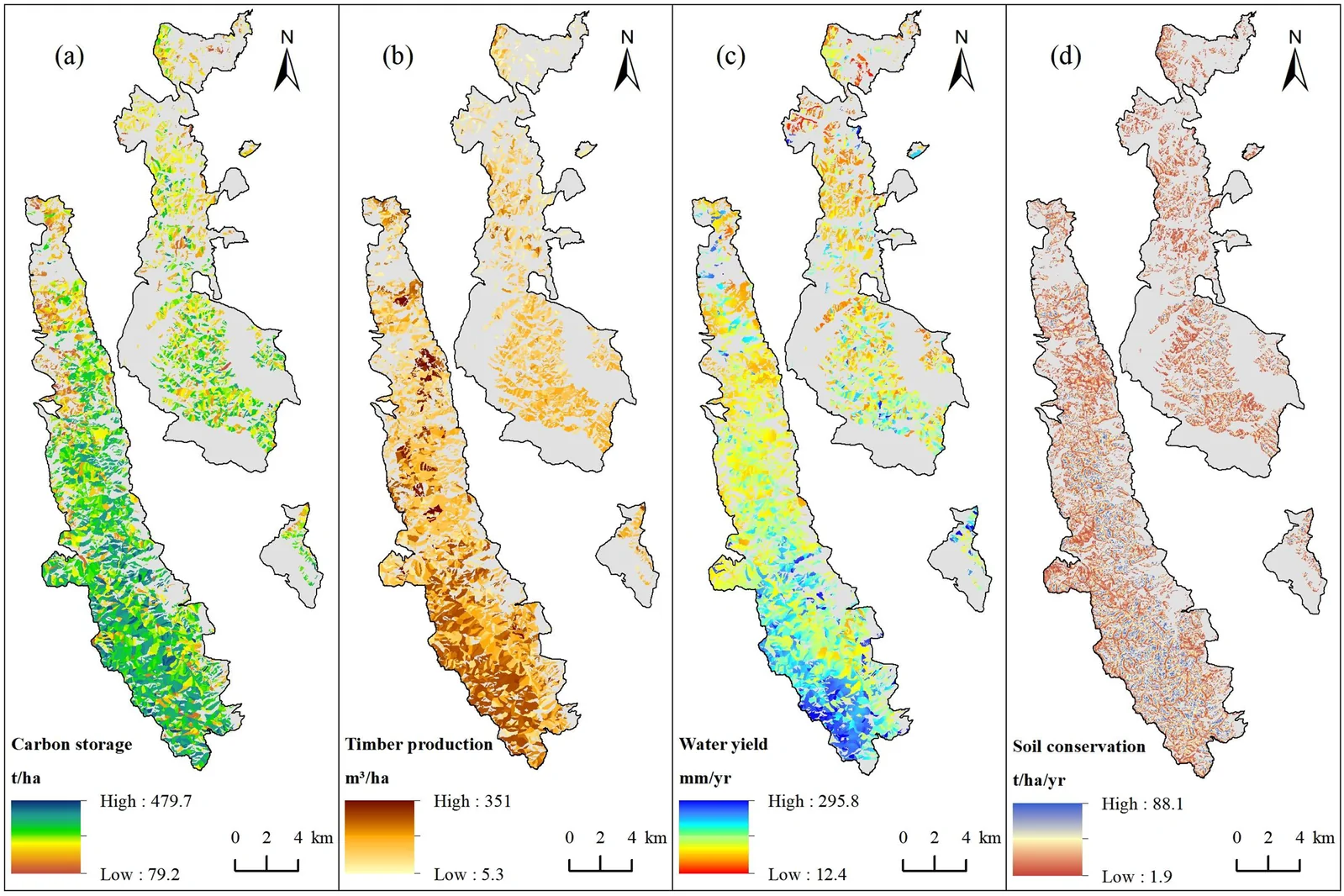
The spatial distribution of carbon storage **(a)**, timber production **(b)**, water yield **(c)**, and soil conservation **(d)**.

### 3.2 Spatial heterogeneity of supply capacity, trade-off and comprehensive benefits for multiple ESs

The SI of natural forests was 0.46, which was 64% higher than that of planted forests (0.28) (**Figure 4**). Among different natural forest types, *Betula platyphylla* forest provided the highest SI, whereas *Quercus wutaishanica* forest, *Pinus armandii* forest, *Populus davidiana* forest, and broad-leaved mixed forest exhibited moderate SI values. Compared to natural forests, the low SI values were observed in all planted forests of *Picea crassifolia* forest, *Larix principis-rupprechtii* forest, and *Pinus tabuliformis* forest, coniferous mixed forest, coniferous and broad-leaved mixed forest (**Figure 5**). In contrast, the TI exhibited no clear spatial clustering across the study region (**Figure 5**), and the average TI of planted and natural forests showed little difference, they were 0.23 and 0.24, respectively (**Figure 4**). High TI values were found in coniferous mixed forest, broad-leaved mixed forest, coniferous and broad-leaved mixed forest, followed by *Picea crassifolia* forest, *Larix principis-rupprechtii* forest, and *Betula platyphylla* forest, whereas *Pinus tabuliformis* forest, *Quercus wutaishanica* forest, *Pinus armandii* forest and *Populus davidiana* forest had low TI values. After incorporating trade-off effects, the CI still exhibited spatial heterogeneous (**Figure 5**), but the difference between natural and planted forests was small, 0.89 for natural forests, only 7% higher than the 0.83 for planted forests (**Figure 4**). Among different forest types, *Betula platyphylla* forest had the highest CI, followed by *Quercus wutaishanica* forest, broad-leaved mixed forest, *Pinus armandii* forest and *Populus davidiana* forest, whereas *Picea crassifolia* forest, *Larix principis-rupprechtii* forest, *Pinus tabuliformis* forest, coniferous and broad-leaved mixed forest had low CI, with coniferous mixed forest showing the lowest value.

**Figure 4:**
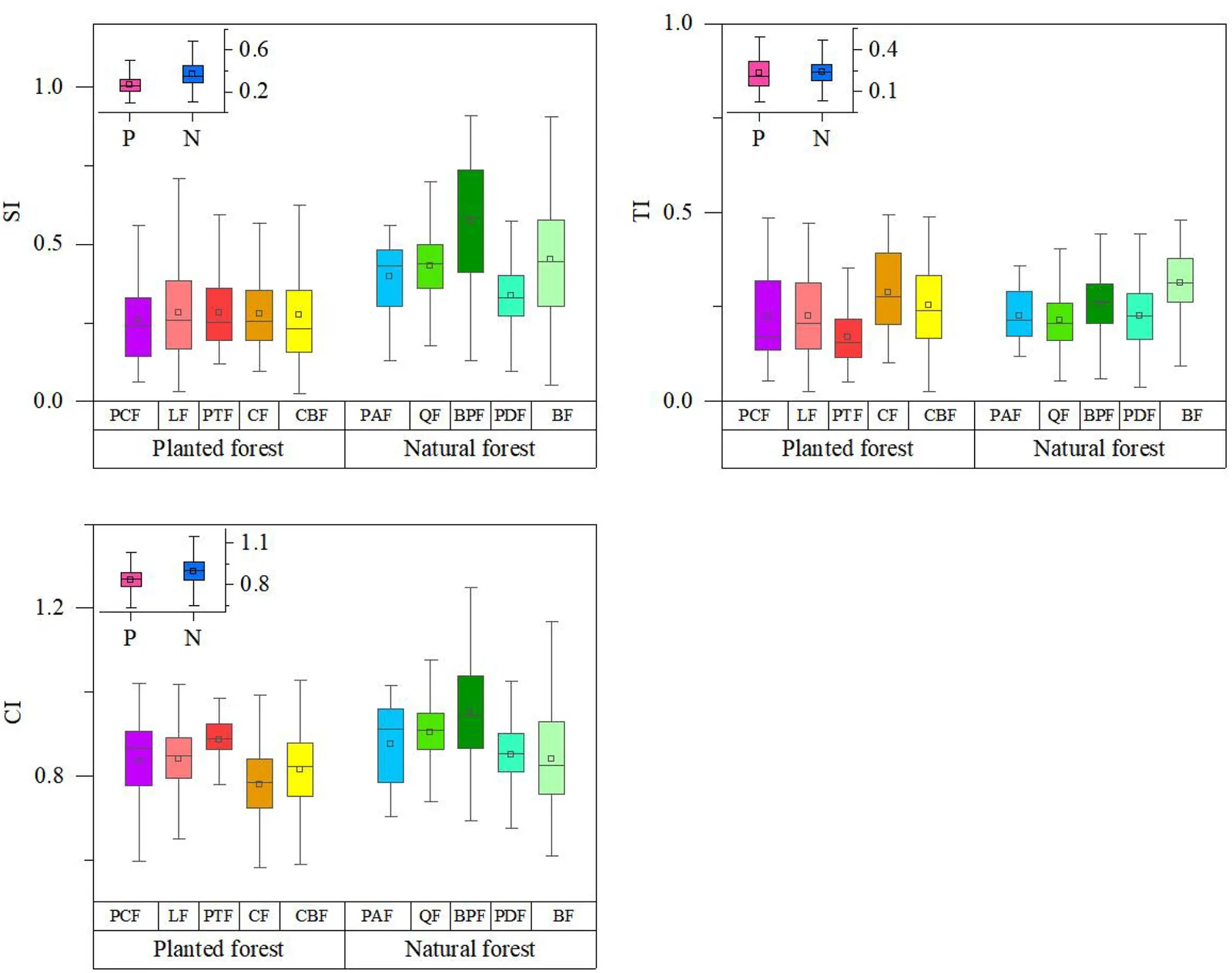
The SI (supply index), TI (trade-off index) and CI (comprehensive index) of different forest types.

**Figure 5:**
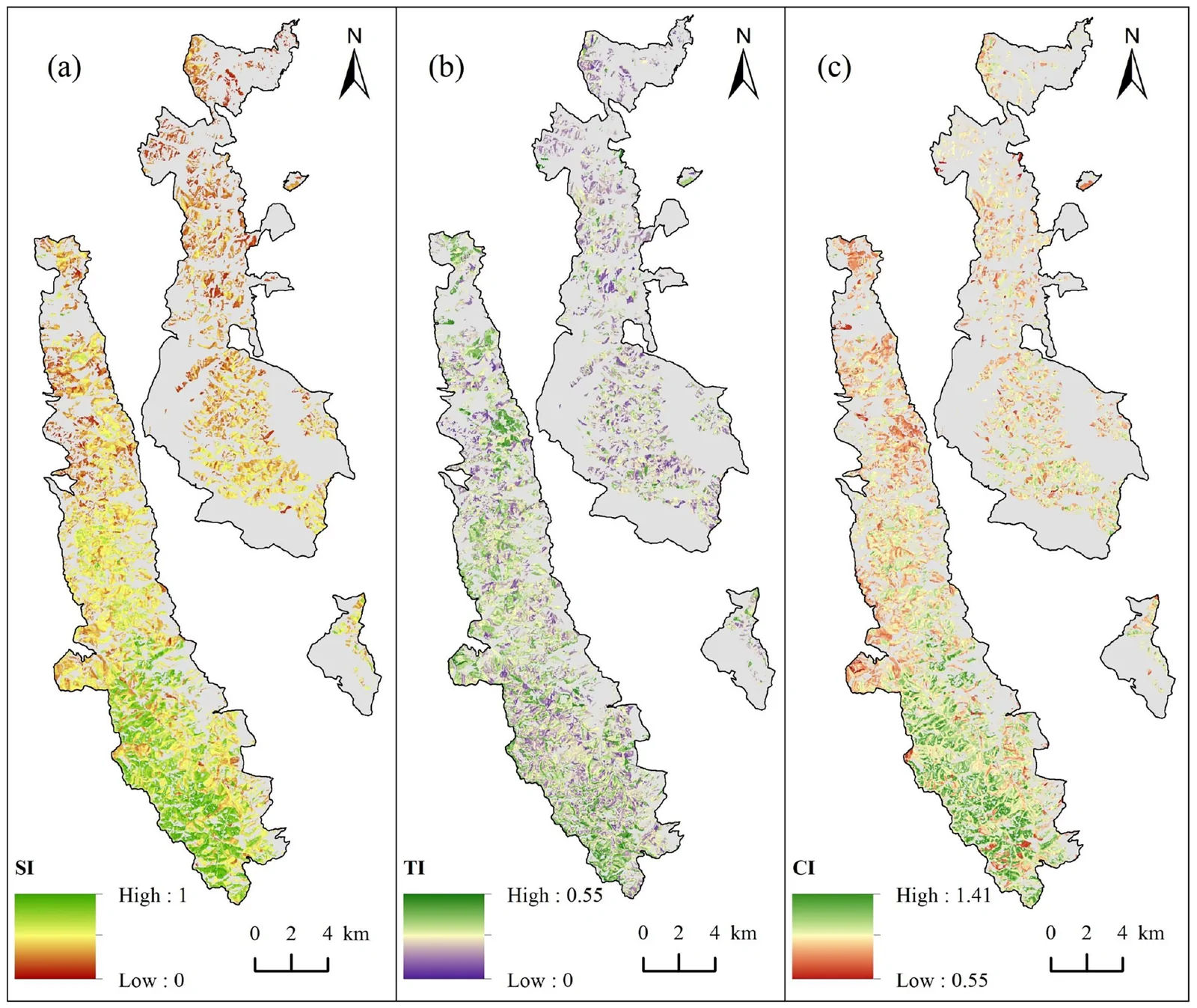
Spatial distribution of supply index **(a)**, trade-off index **(b)** and comprehensive index **(c)**.

### 3.3 The influence of site condition and stand structure on comprehensive benefits for multiple ESs

Correlation analysis revealed that CI was significantly positively correlated with mean annual precipitation, stand age, tree height and DBH, with correlation coefficients of 0.77, 0.84, 0.80, and 0.97 (p< 0.001), respectively (**Figure 6a**). In contrast, CI showed a significant negative correlation with stand density (r = -0.82, p< 0.001). Furthermore, CI was positively correlated with elevation and slope (r = 0.48 and 0.50, respectively) and negatively correlated with mean annual temperature (r = -0.54), all at the 0.01 significance level.

**Figure 6:**
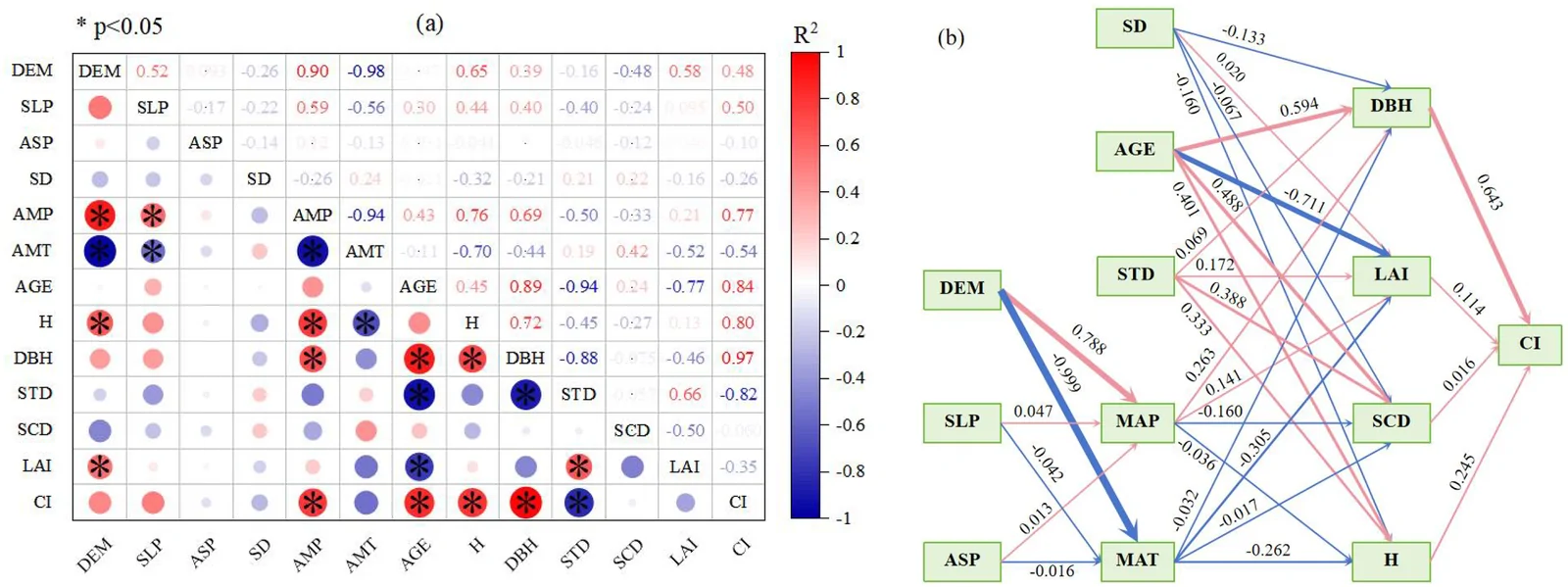
The correlation coefficient and structural equation model of site condition and stand structure on comprehensive index (DEM: elevation; SLP: slope; ASP, aspect; SD, soil thickness; AGE, stand age; STD, stand density; SCD, stand canopy density; MAP, mean annual precipitation; MAT, mean annual temperature; DBH, diameter at breast height; LAI, leaf area index; H, tree height; CI, comprehensive index. The blue color represents negative correlation or effect, and the red color represents positive correlation or effect.).

According to the results of structural equation model (**Figure 6b**), CI was directly influenced by DBH and tree height, their standardized path coefficients were 0.463 and 0.245. DBH was indirectly and positively influenced by stand age and mean annual precipitation, their standardized path coefficients were 0.463 and 0.245. Tree height was directly and positively influenced by stand age and stand density, their standardized path coefficients were 0.401 and 0.333, but negatively influenced by mean annual temperature with a standardized path coefficient of -0.242. Precipitation and temperature, in return, were significantly influenced by elevation. Overall, topography had a significant direct impact on climate conditions, which then together with stand structure affected tree growth and ultimately the CI. Therefore, combining the results of correlation analysis and structural equation model, precipitation, stand age, tree height, DBH, and stand density were selected as the final variables to analyze their constraint relationships with CI.

**Figure 7** shows that the key drivers exhibited strong non-linear relationships with CI. The CI of natural forests was significantly higher than that of planted forests, and their thresholds also differed markedly. Specifically, CI increased with rising mean annual precipitation (**Figure 7a**), with thresholds of 590 mm for planted forests and 651 mm for natural forests. CI also rising with stand age (**Figure 7b**), showing thresholds of 54 years for natural forests and 28 years for planted forests. In response to stand density (**Figure 7c**), CI of natural forests decreased beyond a threshold of 405 trees/ha, whereas CI of planted forests first increased and then decreased, with a threshold of 1,524 trees/ha. With rising tree height (**Figure 7d**), all CI of both natural forests and planted forests first increased and then decreased, with thresholds of 21 m for natural forests and 12 m for planted forests. Regarding DBH (**Figure 7e**), CI of natural forest increased and then stabilized beyond a threshold of 36 cm, while CI of planted forests first increased then decreased with a threshold of 14 cm. In summary, natural forests exhibited higher comprehensive benefits than planted forests, primarily due to their higher precipitation, stand age, tree height, and DBH, as well as their lower stand density.

**Figure 7:**
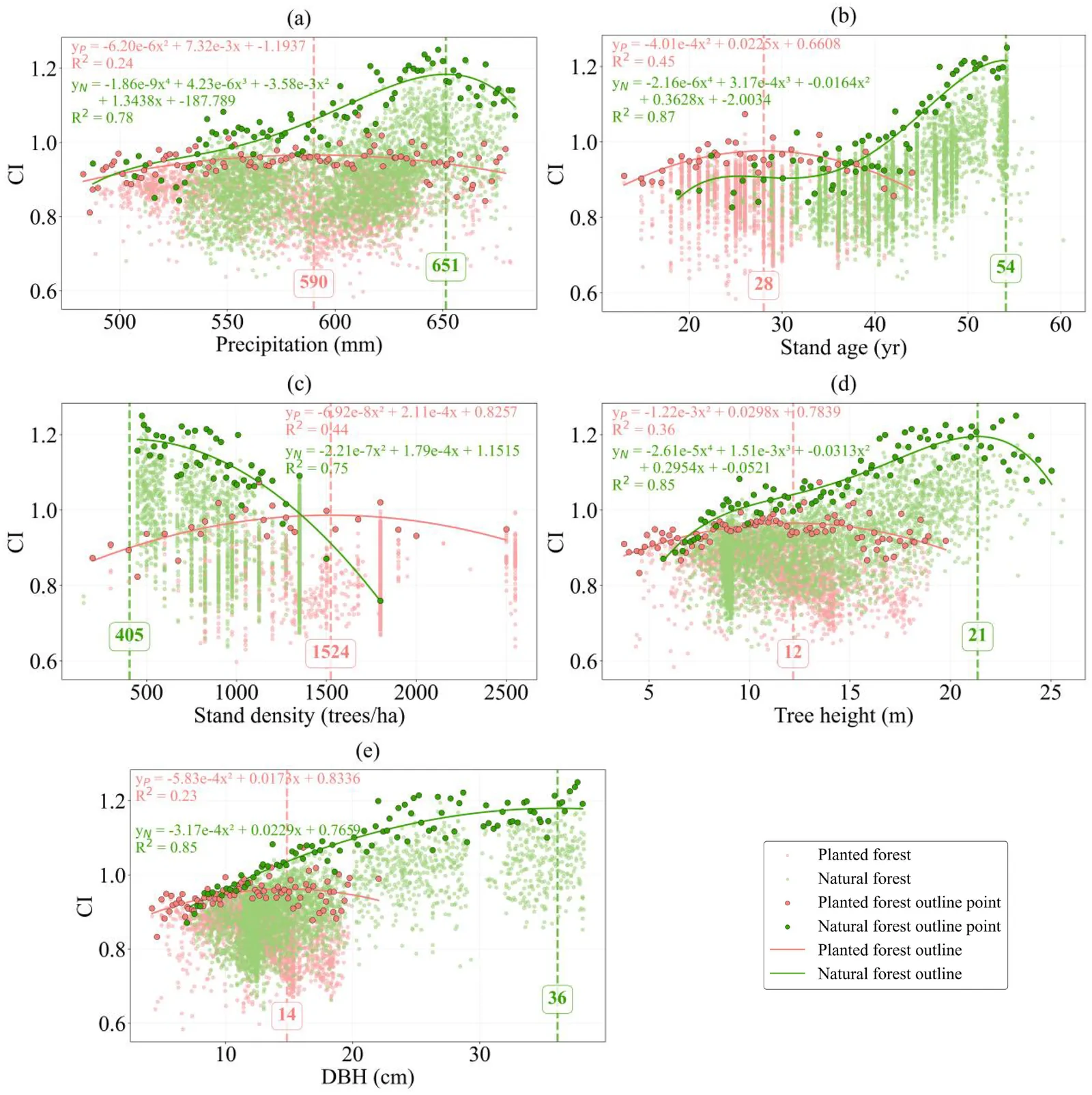
The variation of comprehensive index with precipitation **(a)**, stand age **(b)**, stand density **(c)**, tree height **(d)**, and DBH **(e)**.

## 4 Discussion

### 4.1 Considering the trade-off effect for balanced management of multiple ESs

Among different forest types, *Betula platyphylla* forest exhibited the highest comprehensive benefit, owing to its high supply capacity and high synergy (**Figure 8**). *Quercus wutaishanica* forest, broad-leaved mixed forest, *Pinus armandii* forest, and *Populus davidiana* forest followed, all demonstrating high synergy but with lower supply capacity than *Betula platyphylla* forest. *Picea crassifolia* forest, *Larix principis-rupprechtii* forest, *Pinus tabuliformis* forest, coniferous and broad-leaved mixed forest showed high synergy but low supply capacity, resulting in low comprehensive benefit. Coniferous mixed forest had the lowest synergy and lowest supply capacity, thus ranking lowest comprehensive benefit. In summary, natural forests displayed high comprehensive benefit due to their high supply capacity and moderate synergy, whereas planted forests exhibited low comprehensive benefit because of their low supply capacity and high synergy (**Figure 8**). Additionally, the difference in supply capacity between natural and planted forests was substantial, but the difference in comprehensive benefit became less pronounced after accounting for trade-off effects.

**Figure 8:**
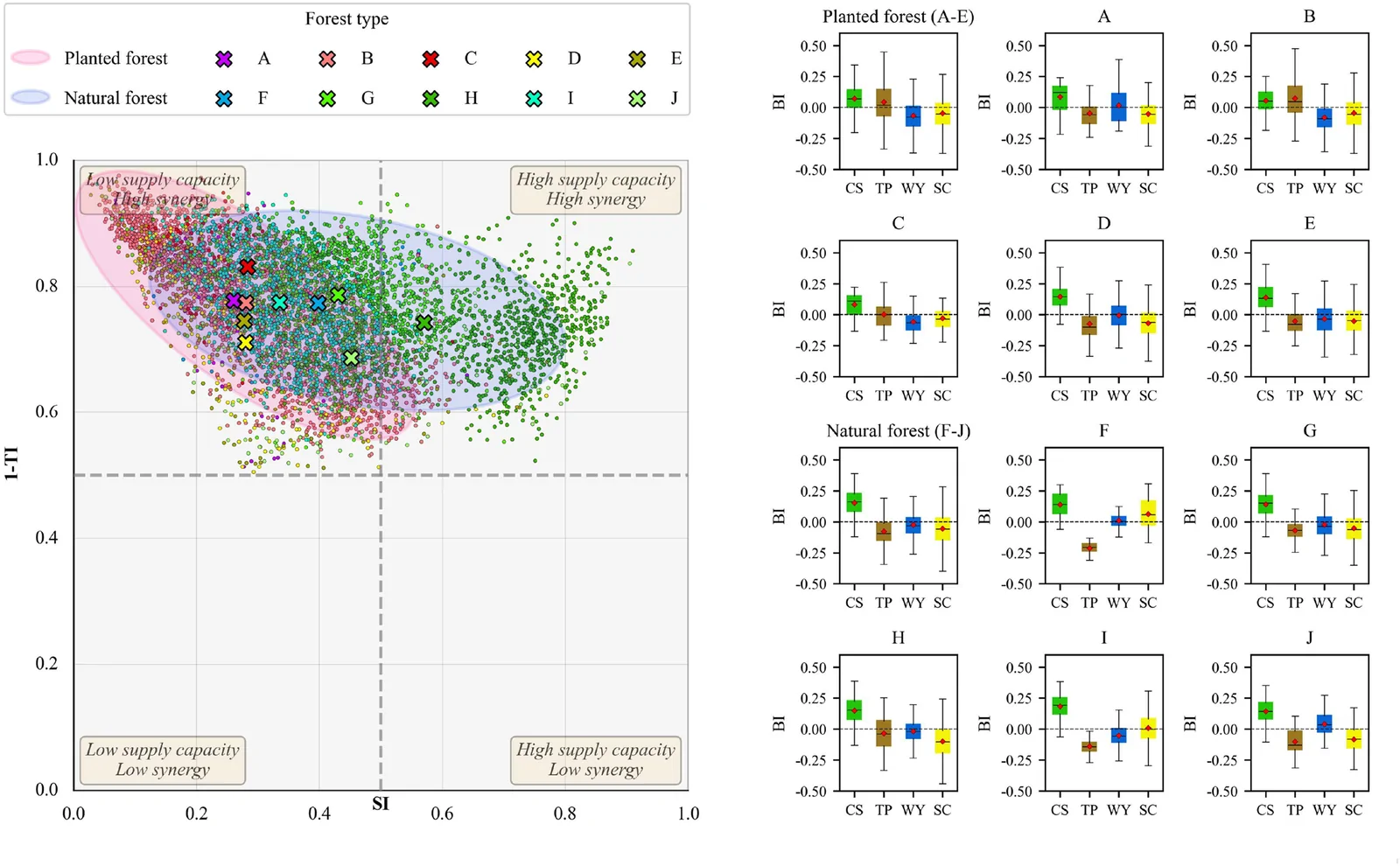
The SI (supply index), TI (trade-off index) and BI (benefit index) of multiple ESs in different forest types [**(A)** *Picea crassifolia* forest; **(B)** *Larix principis-rupprechtii* forest; **(C)** *Pinus tabuliformis* forest; **(D)** Coniferous mixed forest; **(E)** Coniferous and broad-leaved mixed forest; **(F)** *Pinus armandii* forest; **(G)** *Quercus wutaishanica* forest; **(H)** *Betula platyphylla* forest; **(I)** *Populus davidiana* forest; **(J)** Broad-leaved mixed forest; CS, carbon storage; TP, timber production; WY, water yield; SC, soil conservation].

To identify the causes of trade-offs among multiple ESs across different forest types, the benefit index (BI) of each individual ES was evaluated for different forest types (**Figure 8**). The results indicated that planted forests exhibited pronounced positive benefits for carbon storage and timber production service, but negative benefits for water yield service, particularly in *Larix principis-rupprechtii* forest. In contrast, natural forests showed pronounced positive benefits for carbon storage but negative benefits for timber production; notably, *Pinus armandii* forest and *Populus davidiana* forest showed high negative benefit of timber production service. In summary, planted forests were characterized by a marked trade-off between carbon storage, timber production service, and water yield service, whereas natural forests were predominantly characterized by a trade-off between carbon storage and timber production service. These findings suggest that future forest management practices should prioritize enhancing the water yield service of planted forests and the timber production service of natural forests.

### 4.2 Suggestion for zoning and classification of forest management

To better guide forest management practices, the study area should be classified into sub-regions based on CI level, rather than being treated as a single homogeneous region. For each sub-region, we calculated the area proportions of different forest types, site conditions and stand structure gradients, and subsequently proposed specific, science-based management strategies to optimize stand structure (**Figure 9**).

**Figure 9:**
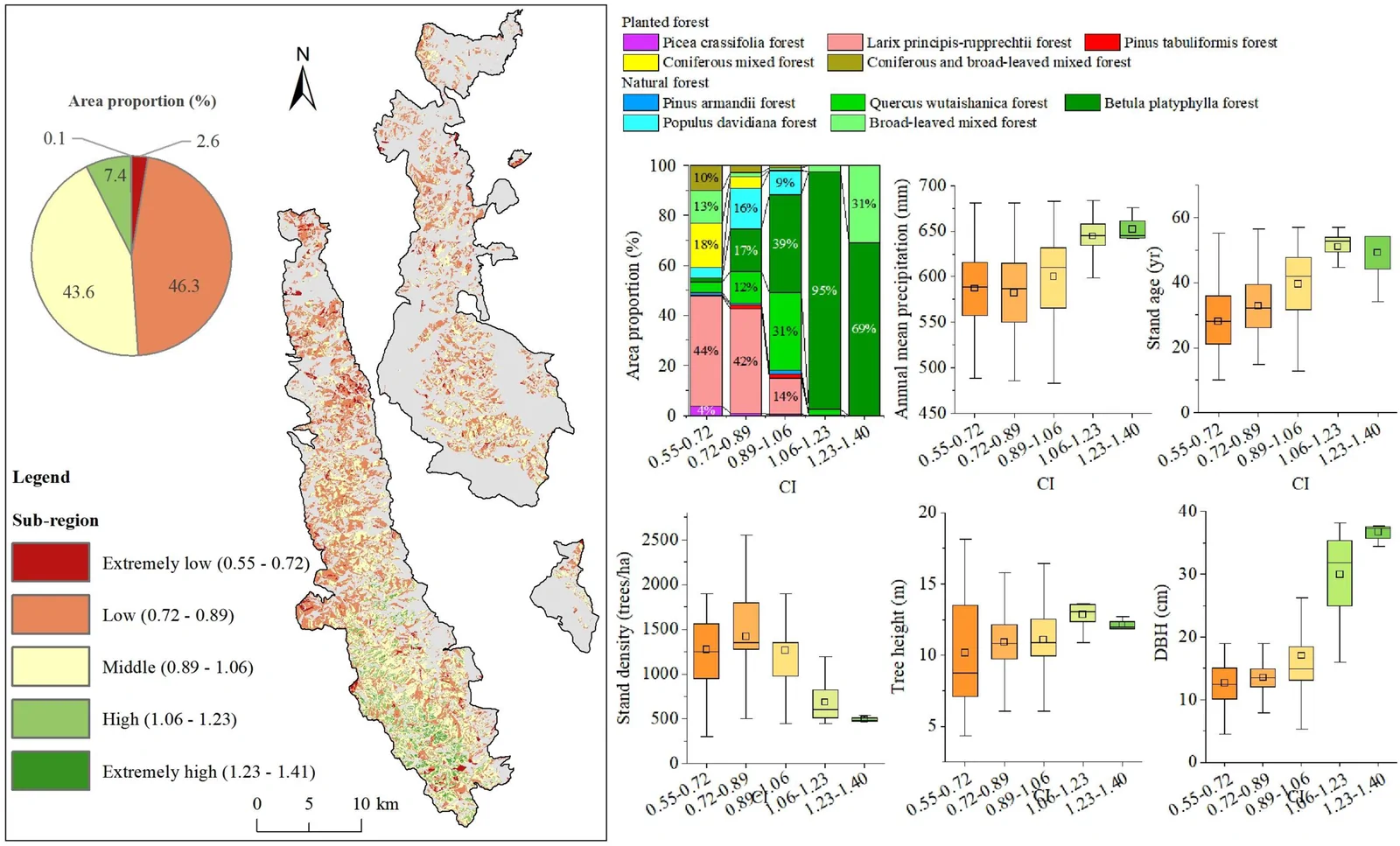
The spatial distribution of different comprehensive index levels and their area proportion of each forest type, and their characteristics of site condition and stand structure.

The results showed that extremely low CI region (with CI of 0.55-0.72) accounted for 2.6% of the total area and was mainly concentrated in the northern part of LPM, where *Larix principis-rupprechtii* forest, coniferous mixed forest, broad-leaved mixed forest, coniferous and broad-leaved mixed forest were predominantly distributed. The low CI region (0.72-0.89) accounted for 46.3% of the total area and was primarily located in the northern and central parts of LPM, with *Larix principis-rupprechtii* forest, *Betula platyphylla* forest, *Populus davidiana* forest and *Quercus wutaishanica* forest being the main forest types. The extremely low and low CI regions were largely occupied by *Larix principis-rupprechtii* forest and were characterized by low precipitation, low stand age, high stand density, and low DBH. For those regions, the comprehensive benefits of multiple ESs should be improved by implementing high-intensity thinning to optimize stand structure, promote DBH growth, and increase the proportion of middle-aged and mature forests.

The middle CI region (0.89-1.06) occupied a large proportion of the total area (43.6%) and was mainly concentrated in the central and southern parts of LPM, where natural forests such as *Betula platyphylla* forest, *Quercus wutaishanica* forest, *Larix principis-rupprechtii* forest, and *Populus davidiana* forest predominated. These regions were characterized by moderate precipitation, stand age, and tree height, but relatively high stand density and low DBH. For this region, our results suggest that light thinning may help optimize stand structure, potentially facilitating the production of the production of large-diameter timber and enhance the comprehensive benefits of multiple ESs.

The high (1.06-1.23) and extremely high (1.23-1.40) CI regions accounted for 7.4% and 0.1% of the total area, respectively. They were mainly concentrated in the southern part of LPM, where natural forests such as *Betula platyphylla* forest and broad-leaved mixed forest dominated. These regions serve as important water source areas and are characterized by high precipitation, mature forest, low stand density, and large DBH. For these regions, forest management may prioritize protection, and thinning may be not necessary and urgent.

### 4.3 The suggestions for forest stand structure optimization

Planted forests have expanded dramatically worldwide. The total global area of planted forests is estimated at 264 million ha (approximately 7% of global forest area), increasing by about 5 million ha annually, and contributing roughly 2/3 of global roundwood production (Dai et al., 2017). China possesses the largest planted forest area and the fastest growth rate globally; however, its plantation productivity remains notably low, with a substantial gap compared to that of developed countries (Zhang et al., 2012; Zhao et al., 2012). Nevertheless, planted forests exhibit pronounced trade-offs among different ESs. For instance, environmental issues such as biodiversity loss and water resource shortages become increasingly conspicuous. Similarly, the results of the present study indicate that planted forests provide marked positive benefits for carbon storage and timber production services, but negative benefits for water yield service (**Figure 8**). Therefore, appropriate forest management strategies such as tending and thinning may be needed to guide the enhancement of synergies among competing ES in planted forests.

Furthermore, our results showed that the maximum CI value of natural forests was significantly higher than that of planted forests (**Figure 7**). Even under similar precipitation and stand age conditions, there is a considerable gap in the CI between planted and natural forests. Therefore, the stand structure of planted forests could be optimized by referencing the structural attributes, particularly stand density of natural forests under similar site conditions and stand ages, thereby enhancing the comprehensive benefits of planted forests through learning from the natural forest analogs. To this end, we quantified the difference in stand density between planted and natural forests (**Figure 10**). The results indicated that stand density in planted forests was generally higher than that in natural forests, especially in young (<20 yr) and middle-aged (21–30 yr) stands. These findings suggest that, under comparable precipitation and stand age conditions, thinning interventions in planted forests could be designed with reference to the stand density levels observed in natural forests. For example, under similar precipitation (≤530 mm) and stand age (≤20 yr) conditions, the stand density of planted forests could be reduced from 1,617 to 1,050 trees/ha, with reference to natural forests. Finally, these prescriptions are based on observational relationships between stand structure and ESs rather than on controlled experiments or simulation, and should therefore be regarded as hypotheses to be tested through field trials or modeling before being applied operationally.

**Figure 10:**
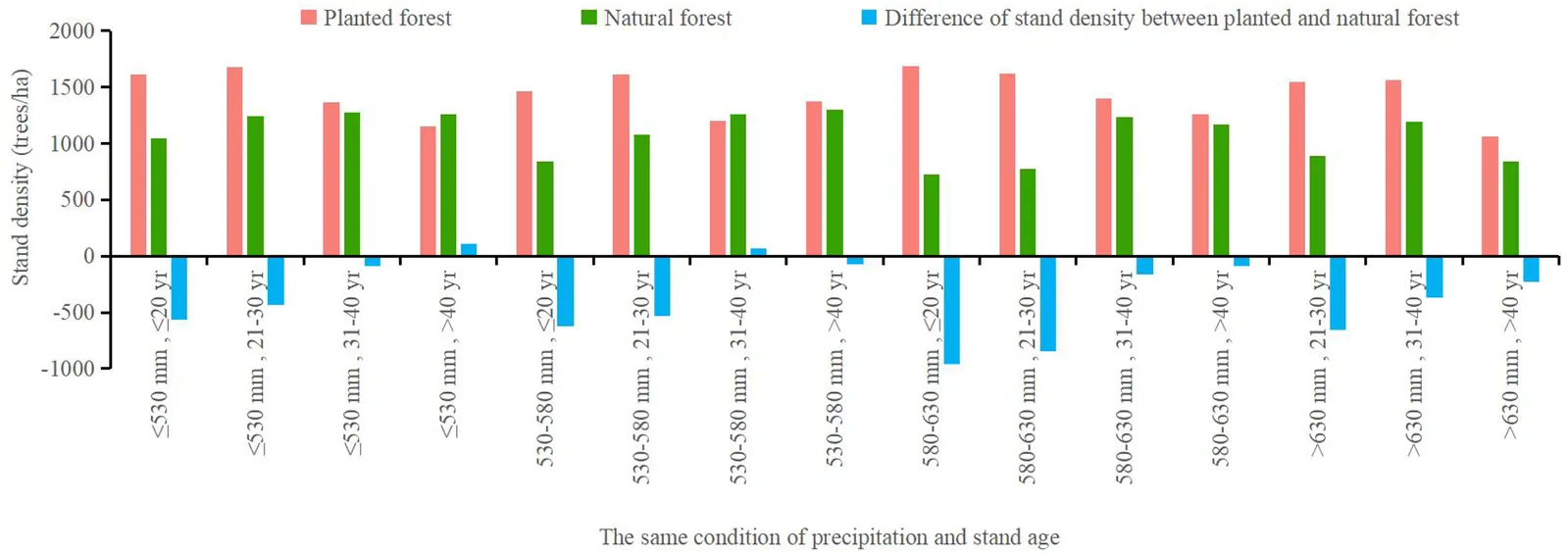
The difference of stand density between planted and natural forests under the same conditions of mean annual precipitation and stand ages.

### 4.4 Limitations of this study and outlook for future research

Several limitations of this study should be acknowledged. First, the thresholds identified in this study are derived from the upper boundary lines of the observed CI-driver relationships and therefore represent constraint-based thresholds, rather than marginal trade-off optima derived from a formal multi-objective optimization. A fully trade-off analysis would require quantifying the marginal gains and losses among individual ecosystem services - that is, the marginal conversion rates along the efficiency frontier - which cannot be obtained from the boundary line approach alone. Accordingly, the thresholds reported here should be interpreted as reference ranges that indicate where the comprehensive benefit can be no longer further improved under given site and structural conditions, rather than as definitive optimal solutions. Second, our analysis is based on observational data and a snapshot assessment; it does not capture the dynamic responses of ESs to management interventions over time. Third, and importantly, a gap remains between the thresholds identified here and their direct application to forest structure optimization. Our analysis identifies the site and structural conditions under which the comprehensive benefit is maximized, but it does not specify how stand structure should be adjusted - through which combinations of thinning intensity, frequency, and timing - to move a given stand toward those conditions. Translating these thresholds into operational silvicultural prescriptions would require additional information on stand dynamics, treatment responses, and the economic and ecological constraints of specific management units. Future work could integrate marginal trade-off analysis, process-based simulation, and stand-level silvicultural experiments to test whether the identified thresholds remain valid under alternative management scenarios and changing environmental conditions, thereby providing a stronger basis for operational forest structure optimization.

Additionally, this study is based on a single forest resource inventory in 2016 and therefore represents a snapshot assessment rather than a dynamic analysis. Forest ecosystems are inherently dynamic: stand age, DBH, density, and species composition change over time and respond jointly to silvicultural treatments. Although forest patches in our study region differ in stand age and structure, allowing us to compare ES supply and trade-offs across stands of different developmental stages, this cross-sectional design captures differences among stands rather than the temporal trajectory of a given stand. Consequently, our analysis cannot project future forest growth or predict how a specific stand will respond to a given silvicultural prescription. The identified thresholds should therefore be interpreted as reference conditions for current stands and as a basis for prioritizing where management may be needed, rather than as prescriptions validated for future conditions. Future work could couple forest growth models or process-based simulation with the threshold framework developed here to project stand development under alternative management scenarios and to test whether the identified thresholds remain valid over time.

In addition, the ESs of timber production and carbon storage in this study were represented by current-state indicators of standing timber volume (m³/ha) and carbon stock (t/ha), rather than by their increments. Strictly speaking, timber production should be quantified by annual increment or harvest data, and carbon sequestration should be quantified by the net change in carbon stock over time (i.e., carbon sequestration rate), because both are flow variables rather than stocks. Using current-state indicators therefore reflects the level or potential of these ESs at a given point in time, but does not capture their dynamics or their response to management interventions. This approach was adopted in this study because our analysis is based on a single forest resource inventory and does not include growth or harvest data, which precludes the calculation of increments over time. Consequently, the timber production and carbon storage results should be interpreted as an assessment of the current supply level of these ESs rather than of their annual production or sequestration rates. Future studies should incorporate repeated inventories, growth models, or process-based simulation to quantify annual timber increment (as timber production service) and net carbon sequestration, which would allow these two functions to be evaluated in terms of their flow rather than their stock, and would provide a more appropriate basis for assessing the effects of forest management on timber production and carbon sequestration.

## 5 Conclusion

We evaluated four key ecosystem services (ESs) in the LPM region and quantified the comprehensive benefits of multiple ESs using a comprehensive index (CI) that integrates the supply index (SI) and trade-off index (TI). We also identified the critical thresholds of site condition and stand structure influencing CI. The main conclusions are as follows: (1) The four individual ESs exhibited high values in the southern part of LPM and low values in the northern part. Natural forests provide higher carbon storage, water yield, and soil conservation services than planted forests, but expect for timber production. (2) The difference in comprehensive benefit between natural and planted forests diminished after accounting for trade-off effects, and this remaining difference was primarily attributable to their local environmental conditions. The water yield service of planted forests and the timber production service of natural forests should be enhanced in future forest management practice. (3) Precipitation, stand age, tree height, and DBH showed significant positive correlations with the comprehensive benefits of multiple ESs, whereas stand density exerted a strong negative impact. All these factors exhibited significant non-linear relationships with critical thresholds. The stand density of planted forests could be optimized through thinning, drawing on natural forest benchmarks under comparable precipitation and stand age conditions. Overall, the findings of this study can provide a reference for developing evidence-based strategies to achieve high comprehensive benefits of multiple ESs at regional scale, which is critical for sustainable forest management practices.

## Data Availability

The original contributions presented in the study are included in the article/supplementary material. Further inquiries can be directed to the corresponding authors.
